# Associations among Physician–Patient Communication, Patient Satisfaction, and Clinical Effectiveness of Overactive Bladder Medication: A Survey of Patients with Overactive Bladder

**DOI:** 10.3390/jcm11144087

**Published:** 2022-07-14

**Authors:** Naoko Izumi, Tomohiro Matsuo, Yoshihisa Matsukawa

**Affiliations:** 1 Medical Affairs, Internal Medicine, Pfizer Japan Inc., 3-22-7 Yoyogi, Shibuya-ku, Tokyo 151-8589, Japan; 2 Department of Urology, Nagasaki University Graduate School of Biomedical Sciences, Nagasaki 852-8501, Japan; tomo1228@nagasaki-u.ac.jp; 3 Department of Urology, Nagoya University Graduate School of Medicine, Nagoya 466-8550, Japan; yoshi44@med.nagoya-u.ac.jp

**Keywords:** urinary bladder, overactive, communication, medication adherence, physician–patient relations, surveys and questionnaires

## Abstract

This cross-sectional survey study evaluated associations between physician–patient communication and patient satisfaction with overactive bladder (OAB) medical care or clinical effectiveness. Japanese patients aged ≥50 years with OAB who had taken OAB medication within 2 years received a web-based survey regarding OAB medical care, physician–patient communication on OAB symptoms and treatment, and OAB symptom score (OABSS) change with treatment. Associations between physician–patient communication and patient satisfaction or OAB medication effectiveness (≥3-point improvement in OABSS) were investigated by multivariate analysis with confounding factors. Stratified analyses were performed based on medication continuation or discontinuation (for reasons except symptom improvement). Of the 1004 respondents included in the analyses, 58.0% continued treatment, and 23.7% discontinued for reasons except symptom improvement. Satisfaction with OAB care was associated with reported effectiveness, medication side effects, physician–patient communication, and whether medication was switched. Medication effectiveness was associated with patient–physician communication, female sex, and not switching medication. Significantly more patients in the continuation group were satisfied and had improvement of ≥3 points in OABSS (*p* < 0.001 for both). The findings suggest that, in Japan, adequate physician–patient communication contributes significantly to improving clinical effectiveness and satisfaction with medical care in OAB patients as well as treatment continuation.

## 1. Introduction

Patients with overactive bladder (OAB), a dysfunction of the lower urinary tract, experience urinary urgency, usually accompanied by increased daytime frequency and nocturia, with/without urinary incontinence [1]. Population-based studies report a range in OAB prevalence of 7–27% in men and 9–43% in women [2]. An epidemiological survey estimated that 12.4% of the Japanese population aged ≥40 years had OAB [3].

OAB is a symptomatic syndrome, and there are few objective tests available in routine practice [2]. Diagnosis and treatment decisions are most often based on patient-reported symptoms, which are subjective. Therefore, physician–patient communication seems to be important.

Patient surveys have found that patients value communication with physicians; however, patients prefer that physicians initiate conversations related to OAB symptoms, as they find initiating such discussions embarrassing [4,5]. Unfortunately, the same surveys reported that physicians are rarely the initiators, and patients are dissatisfied with the quantity and quality of communication they receive [4,5].

Medication persistence rates are lower for OAB than for chronic diseases, ranging from 28% at 6 months [6] to 12–25% at 1 year [6,7]. In general, poor physician communication increases the risk of poor treatment adherence in patients [8]. Significantly higher levels of treatment adherence were reported for OAB patients with more frequent communication with their physician versus those with less frequent communication [4]. Poor physician–patient communication may interfere with physicians’ ability to fully understand a patient’s symptoms and condition. Thus, appropriate treatment planning and achievement of treatment goals may be hindered.

We hypothesized that physician–patient communication is associated with patient satisfaction with medical care for OAB and its effectiveness. The objective of this cross-sectional survey study was to examine whether there is an association between patient satisfaction with medical care or clinical effectiveness and the level of communication between physicians and patients receiving medication for OAB.

## 2. Methods

### 2.1. Study Population

Patients were included if they were aged ≥50 years, diagnosed with OAB based on screening questions, had taken OAB medication in the past 2 years (discontinuation was allowed), and consented to the survey. Patients who used transdermal patches containing oxybutynin hydrochloride were excluded because communications about this drug delivery method would likely differ from explanations of other OAB medication drug delivery methods, which may affect the results of this study. Patients who did not meet the OAB symptom score (OABSS) [9] criteria for OAB (<2 points for urinary urgency or a total score of <3 points) immediately prior to starting medication were also excluded.

### 2.2. Study Design

This cross-sectional survey was conducted in October–November 2020 using data from an Internet panel (Macromill Carenet Inc., Tokyo, Japan). The panel is composed of approximately 1,300,000 voluntary registrants located throughout Japan. The Automatic Internet Research System (Macromill Carenet Inc.) was used to administer the surveys.

**Figure 1:**
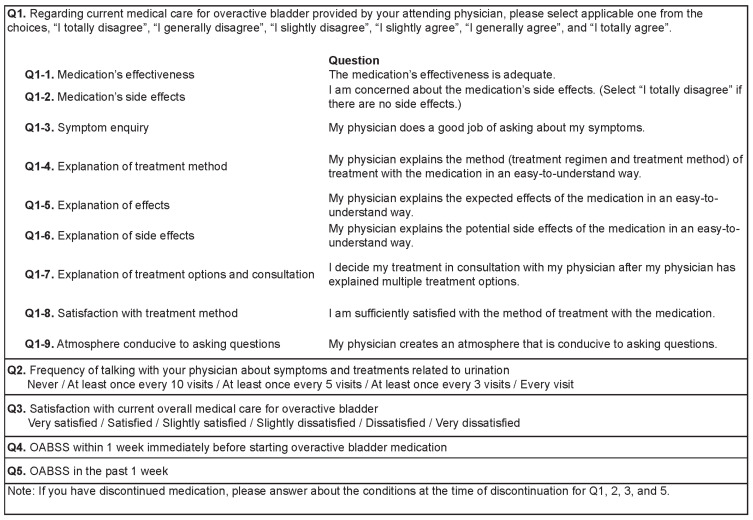
Survey questions. OABSS, overactive bladder symptom score.

Eligible patients completed a web-based survey that consisted of five questions regarding their current physician-provided medical care for OAB (Q1, which included nine parts), the frequency at which they discussed symptoms and treatments related to urination with their physician (Q2), their level of satisfaction with their current OAB medical care (Q3), and their OABSS within 1 week prior to starting OAB medication (Q4) and in the past week (Q5) (Figure 1). Patients who had discontinued their OAB medication were asked to respond to survey items based on the time of discontinuation (Q1, Q2, Q3, and Q5).

This study was conducted in accordance with the Ethical Guidelines for Medical and Health Research Involving Human Subjects issued by Japanese regulatory authorities [10]. This study did not require institutional review board or independent ethics committee approval because the guidelines do not require such approval for information that has already been anonymized.

### 2.3. Study Endpoints

This study had two endpoints. Endpoint 1 was to determine whether there were associations among the effectiveness of OAB medication, medication side effects, physician–patient communication, and satisfaction with overall OAB treatment. Endpoint 2 was to determine whether there was an association between physician–patient communication and the effectiveness of OAB medication, determined by the presence or absence of a ≥3-point improvement in OABSS (regarded as clinically significant [11]) between the week immediately prior to initiating medication and the week the patient discontinued medication or completed the survey.

### 2.4. Statistical Analysis

No power calculation was performed to determine the sample size for this descriptive survey. The target number of respondents was ≤1000. Patients who completed the survey and had been examined at least once after starting OAB medication by the attending physician in charge of OAB medical care were included in the analysis.

Endpoints 1 and 2 were assessed by multivariate analysis using a logistic regression model with confounding factors. For endpoint 1, the response variable was Q3: “Satisfied: Very satisfied, Satisfied, Slightly satisfied” or “Dissatisfied: Slightly dissatisfied, Dissatisfied, Very dissatisfied”. The explanatory variables were respective items of Q1 and Q2. The following explanatory variables were handled as binary variables: for Q1, “I agree: I slightly agree, I generally agree, I totally agree” and “I disagree: I totally disagree, I generally disagree, I slightly disagree”; for Q2, “I have a talk: At least once every 10 visits, At least once every 5 visits, At least once every 3 visits, Every visit” and “Never”. For endpoint 2, the response variable was the presence or absence of improvement in OABSS of ≥3 points. The explanatory variables were respective items of Q1 and Q2.

Stratified analyses were performed for both endpoints using the medication discontinuation group, which was defined as patients who discontinued taking OAB medications for reasons other than symptom improvement, and the continuation group, which comprised patients who continued taking OAB medications. Additionally, the following items were examined using the cross-tabulation chi-square test for the “I agree” and “I disagree” groups in Q1, the “I have a talk” and “Never” groups in Q2, “Satisfied” and “Dissatisfied” groups in medical care satisfaction, and the presence or absence of a ≥3-point improvement in OABSS: presence or absence of OAB medication switch, medication discontinuation and continuation groups, “Medication discontinuation × short-term group (<3 months)” and “Medication continuation × short-term group (<3 months)”, and “Medication discontinuation × short-term group (<3 months)” and “Medication discontinuation × long-term group (≥3 months)”.

A *p*-value of <0.05 was considered statistically significant. Statistical analyses were performed using Python 3.7 (Python Software Foundation, Wilmington, DE, USA).

## 3. Results

### 3.1. Patients

Approximately 49,000 panel registrants were invited to participate in the survey, of which 1314 met the screening criteria. Of these, 1026 (78.1%) met the OAB criteria, and 288 (21.9%) were excluded as non-OAB. In total, 1004 patients were examined at least once after starting OAB medication by the attending physician in charge of OAB medical care and were included in the analysis. Patient characteristics are shown in Table 1. The mean ± standard deviation (SD) age was 70.3 ± 7.1 years. There were 413 (41.1%) female patients; women were significantly younger than men (66.8 ± 6.6 and 72.7 ± 6.4 years, respectively; *p* < 0.001). OAB medication was continued by 582 (58.0%) patients and discontinued by 422 (42.0%); 238 (23.7%) patients discontinued for reasons other than symptom improvement.

### 3.2. Survey Responses

**Figure 2:**
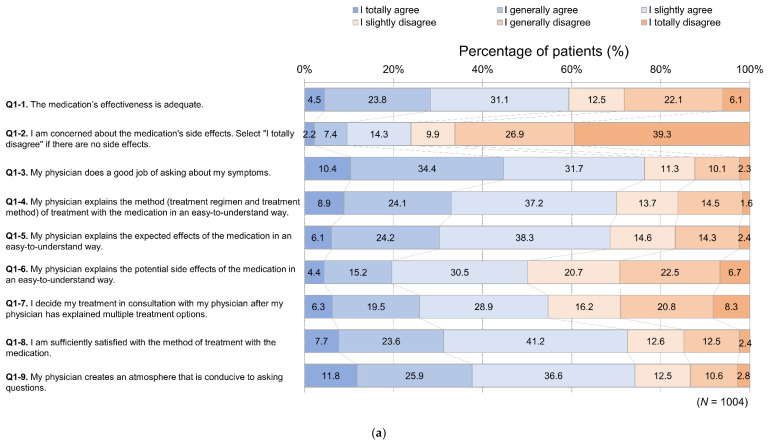

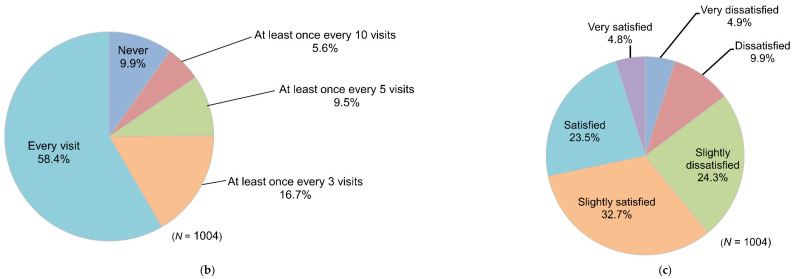
Results of survey questions (**a**) (Q1): Effectiveness, side effects of medication, and communication with physician for overactive bladder medical care; (**b**) (Q2): frequency of talking with your physician about symptoms and treatments related to urination; and (**c**) (Q3): satisfaction with current overall medical care for overactive bladder.

Responses regarding current care for OAB (Q1) are shown in Figure 2a. The most common frequency at which patients talked with physicians about symptoms and treatments related to urination (Q2) was “Every visit” (58.4%); 9.9% responded “Never” (Figure 2b). Cronbach’s alpha for Q1 and Q2 was 0.86. Regarding medical care satisfaction (Q3), 61.0% of patients were satisfied (“Very satisfied”, “Satisfied”, “Slightly satisfied”) (Figure 2c).

### 3.3. Endpoints

The results for endpoint 1 are shown in Table 2. Satisfaction with overall medical care for OAB was associated with effectiveness (*p* < 0.001), concern with medication side effects (*p* < 0.001), communication with physicians (“Q1-6. Explanation of side effects” *p* = 0.02; “Q1-8. Satisfaction with treatment method” *p* < 0.001; “Q1-9. Atmosphere conducive to asking questions” *p* < 0.001), and the presence or absence of medication switch (*p* = 0.045). Effectiveness was most strongly associated with satisfaction. 

The results for endpoint 2 are shown in Table 3. Clinical effectiveness of OAB medication was associated with patient–physician communication (“Q1-3. Symptom enquiry” *p* < 0.001; “Q1-8. Satisfaction with treatment method” *p* < 0.001), “Sex (female)” (*p* < 0.001), and “No medication switch” (*p* = 0.002).

The absence of medication change was associated with both satisfaction and effectiveness. In the group that did not switch medication, the proportions of patients who answered “I agree” for “Q1-1. Medication’s effectiveness”, who were classified into the “satisfied group”, and who had “improvement of ≥3 points in OABSS” were significantly higher (*p* < 0.001, *p* = 0.002, and *p* = 0.02, respectively) than those in the group that switched medication; the proportions of patients who answered “I agree” to “Q1-2. Medication’s side effects” and “Q1-7. Explanation of treatment options and consultation” were significantly lower (*p* = 0.003 and *p* = 0.03, respectively).

### 3.4. Stratified Analyses in the Medication Discontinuation Group

There was no difference in the mean ± SD OABSS prior to initiating OAB medication between the continuation group (9.0 ± 2.6) and the discontinuation group (patients who discontinued for reasons other than symptom improvement; 9.1 ± 2.5, *p* = 0.758). The mean ± SD change in OABSS was significantly greater in the continuation group (3.2 ± 2.8) than in the discontinuation group (1.7 ± 2.3, *p* < 0.001).

In the stratified analysis (Table 4), significantly associated items for endpoint 1 among patients in the discontinuation group were “Q1-1. Medication’s effectiveness” (*p* < 0.001), “Q1-2. Medication’s side effects” (*p* = 0.04), and “Q1-6. Explanation of side effects” (*p* = 0.006). Those among patients in the continuation group were “Q1-1. Medication’s effectiveness” (*p* < 0.001), “Q1-2. Medication’s side effects” (*p* < 0.001), “Q1-8. Satisfaction with treatment method” (*p* < 0.001), “Q1-9. Atmosphere conducive to asking questions” (*p* = 0.01), and “No medication switch” (*p* = 0.04). For endpoint 2, there were no significantly associated items among patients in the discontinuation group; significantly associated items among patients in the continuation group were “Q1-3. Symptom enquiry” (*p* = 0.001), “Q1-8. Satisfaction with treatment method” (*p* = 0.004), “Sex (female)” (*p* = 0.002), “Duration of medication exposure ≥3 months” (*p* = 0.002), and “No medication switch” (*p* = 0.006). For both endpoints 1 and 2, the number of associated items was greater in the continuation group than in the discontinuation group.

**Figure 3:**
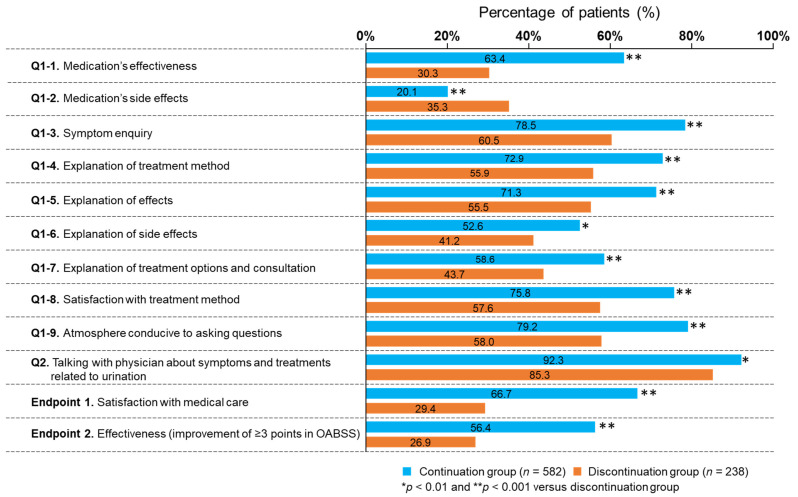
Comparison between patients who continued and those who discontinued overactive bladder medication. OABSS, overactive bladder symptom score.

The continuation group showed significantly better results than the discontinuation group for all items in Q1 and for Q2 as demonstrated by the higher proportion of patients who were satisfied and who had an improvement of ≥3 points in OABSS (*p* < 0.01 for all) (Figure 3).

When comparing the “Discontinuation × short-term group” and “Continuation × short-term group”, the latter group showed significantly better results in satisfaction (*p* < 0.001), improvement of ≥3 points in OABSS (*p* = 0.02), and in all items of Q1 except for “Q1-6. Explanation of side effects” (*p* < 0.001–*p* = 0.0498). When comparing the “Discontinuation × short-term group” and “Discontinuation × long-term group”, no difference was observed in satisfaction or improvement of ≥3 points in OABSS. However, the “Discontinuation × short-term group” had significantly lower proportions of patients who answered “I agree” or “I have a talk” to “Q1-3. Symptom enquiry” (*p* = 0.004), “Q1-4. Explanation of treatment method” (*p* = 0.048), “Q1-7. Explanation of treatment options and consultation” (*p* = 0.001), and “Q2. Frequency of talking with physicians about symptoms and treatments related to urination” (*p* = 0.004) than the “Discontinuation × long-term group”.

## 4. Discussion

This survey showed that medical care satisfaction among OAB patients was associated with treatment effectiveness, side effects, and extent of communication with physicians; clinical effectiveness was also associated with communication. These findings provide clinicians who treat OAB patients with clear evidence that good communication may improve clinical effectiveness and satisfaction with medical care in these patients.

Communication-related items associated with satisfaction were “Q1-9. Atmosphere conducive to asking questions”, “Q1-8. Satisfaction with treatment method”, and “Q1-6. Explanation of side effects”. Previous studies have reported that, for reasons such as embarrassment, OAB patients hesitate to discuss their symptoms with physicians [4,5]. Together, these findings suggest that satisfactory treatment of OAB involves discussion of patient symptoms and that it is important for physicians to create an atmosphere conducive to asking questions. A previous study indicated that physician–patient communication was rather physician-centered, reporting that 83% of questions from physicians were closed-ended and did not encourage patients to discuss their symptoms and treatments [12]. The impact of OAB on quality of life and concerns about/adherence to medication were more frequently addressed when physicians communicated with open-ended questions [12], which may encourage patients to volunteer information. Our study also suggested that satisfied patient understanding of treatment methods improved satisfaction with medical care although this may be associated with providing an atmosphere conducive to asking questions. It is also important for physicians to explain treatment side effects, as OAB medication, particularly anticholinergic agents, may cause dry mouth, constipation, or other reactions in the early stages of treatment [13].

Items associated with a clinically significant improvement [11] of ≥3 points in OABSS were “Q1-3. Symptom enquiry”, “Q1-8. Satisfaction with treatment method”, “Sex (female)”, and “No medication switch”. Understanding subjective symptoms reported by patients is important for treating OAB; thus, listening to patients describe their symptoms and providing appropriate treatment accordingly are considered highly effective management approaches. Additionally, given that patient–physician communication is important for medication adherence [7] and that continuation of treatment is important to achieve an adequate treatment effect, good communication likely affects patient satisfaction with treatment. Improvement in mean change in OABSS was approximately 1 point higher in female than in male patients, suggesting a substantial impact of sex differences on improved effectiveness in terms of OABSS. Almost half of male patients had benign prostatic hyperplasia (46.9%), which may have impacted effectiveness.

“No medication switch” was associated with satisfaction and effectiveness. Between-group (switch or no switch) comparison suggested that not switching medication because the first prescribed medication provided sufficient effectiveness without noticeable side effects may have positively affected satisfaction and effectiveness although this was not formally tested.

The reported OAB medication continuation rate over a 1-year period is low (approximately 30%), and many patients discontinue within a short time after initiation [6,7]. We found a greater number of associated items for both endpoints 1 and 2 (satisfaction and effectiveness) in the continuation group than in the discontinuation group. Additionally, the continuation group showed significantly better results than the discontinuation group in effectiveness, side effects, all items related to communication, satisfaction, and improvement of ≥3 points in OABSS. These findings suggest that both medication effectiveness and communication are important for medication continuation. Our comparisons of the “Discontinuation × short-term group” with the “Continuation × short-term group” or “Discontinuation × long-term group” suggest that medication discontinuation within a short period of time is associated with effectiveness, side effects, satisfaction, and poor communication.

Several clinical studies of OAB medication through 12 weeks of treatment have demonstrated increasing effectiveness over time [14,15,16], which indicates that medication should be continued for at least 3 months to achieve adequate benefit. The results of the present study suggest that communication is important to prevent rapid discontinuation and to improve adherence rates to achieve adequate effectiveness. Communication strategies such as clearly defining treatment goals and increasing education may increase treatment compliance [17]. Patient utilization of a navigation pathway, which has been shown to increase communication, can significantly improve patient retention rates [18].

Study limitations include the online survey design, potentially biasing the study population to include a greater proportion of patients with a high level of web literacy, and the inclusion of only Japanese patients, limiting the generalizability of our findings. Answers about the past may also have been influenced by recall bias. Finally, clinical effectiveness was assessed subjectively by the patient, not objectively. Although patient–physician communication is clearly important, further studies are needed to determine effective physician education and training methods and understand their effect on enhancing patient communication.

## 5. Conclusions

In conclusion, communication with physicians was associated with medical care satisfaction and clinically significant improvement in OABSS among OAB patients in Japan. Furthermore, communication was associated with medication continuation. In particular, communication was considered important for medication continuation during early treatment. The results also suggest that adequate communication can significantly contribute to improving clinical effectiveness and satisfaction with medical care in OAB patients in Japan as well as medication continuation.

## Figures and Tables

**Table 1:** Patient characteristics.

	Total Population *N* = 1004	
Sex		
Male	591	(58.9)
Female	413	(41.1)
Age, years		
Mean (SD)	70.3	(7.1)
Median (IQR)	71.0	(10.0)
<70 years	385	(38.3)
≥70 years	619	(61.7)
OABSS prior to medication		
Mean (SD)	9.0	(2.6)
Median (IQR)	9.0	(4.0)
Change in OABSS		
Mean (SD)	3.1	(2.9)
Median (IQR)	3.0	(4.0)
Clinically significant improvement in OABSS		
Unimproved (<3 points)	471	(46.9)
Improved (≥3 points)	533	(53.1)
Medication adherence		
Continued	582	(58.0)
Discontinued	422	(42.0)
Patient-reported reason for discontinuation		
Symptom improvement	184	(43.6)
No symptom improvement	116	(27.5)
Worsening symptoms	2	(0.5)
Side effects	46	(10.9)
Used on an as needed basis	63	(14.9)
Other	42	(10.0)
Discontinued for any reason other than symptom improvement	238	(23.7)
Discontinued due to symptom improvement	184	(18.3)
Duration of medication exposure		
<1 month	70	(7.0)
1 to <3 months	141	(14.0)
3 to <6 months	141	(14.0)
6 to <12 months	169	(16.8)
≥12 months	483	(48.1)
Switched medication		
Yes	387	(38.5)
No	617	(61.5)
Department consulted		
Urology	733	(73.0)
Internal medicine	240	(23.9)
Other	26	(2.6)
Unknown	5	(0.5)
Type of medical facility visited		
Hospital	391	(38.9)
Clinic	608	(60.6)
Other	5	(0.5)
Comorbidities		
Any	730	(72.7)
Hypertension	413	(41.1)
Dyslipidemia (hyperlipidemia)	193	(19.2)
Diabetes mellitus	141	(14.0)
Benign prostatic hyperplasia (male patients)	277	(46.9)
Other	114	(11.4)
None of the above	274	(27.3)

Data are presented as *n* (%) unless otherwise noted. IQR, interquartile range; OABSS, overactive bladder symptom score; SD, standard deviation.

**Table 2:** Endpoint 1: Association with satisfaction with overall medical care for overactive bladder.

Explanatory Variables	*p*-Value	OR	95% CI
Q1-1. Medication’s effectiveness	<0.001	7.99	(5.59–11.43)
Q1-2. Medication’s side effects	<0.001	0.36	(0.24–0.53)
Q1-3. Symptom enquiry	0.56	1.16	(0.70–1.92)
Q1-4. Explanation of treatment method	0.26	1.34	(0.81–2.24)
Q1-5. Explanation of effects	0.37	1.25	(0.76–2.07)
Q1-6. Explanation of side effects	0.02	1.61	(1.07–2.44)
Q1-7. Explanation of treatment options and consultation	0.06	1.47	(0.98–2.19)
Q1-8. Satisfaction with treatment method	<0.001	2.20	(1.43–3.38)
Q1-9. Atmosphere conducive to asking questions	<0.001	2.50	(1.56–4.01)
Q2. Talking with physician about symptoms and treatments related to urination at least once every 10 visits	0.23	1.44	(0.79–2.63)
Age (≥70 years old)	0.75	0.94	(0.63–1.40)
Sex (female)	0.44	1.17	(0.78–1.76)
Duration of medication exposure is ≥3 months	0.20	0.75	(0.49–1.16)
Have not switched to another type of overactive bladder medication since starting to take it	0.045	1.44	(1.01–2.05)

Chi-square probability: <0.001; Hosmer–Lemeshow *p*-value: 0.28; correct discrimination rate: 0.80. CI, confidence interval; OR, odds ratio.

**Table 3:** Endpoint 2: Association with the effectiveness of overactive bladder medication (presence or absence of improvement ≥3 points in OABSS).

Explanatory Variables	*p*-Value	OR	95% CI
Q1-3. Symptom enquiry	<0.001	2.45	(1.62–3.70)
Q1-4. Explanation of treatment method	0.18	1.34	(0.87–2.06)
Q1-5. Explanation of effects	0.70	0.92	(0.60–1.41)
Q1-6. Explanation of side effects	0.80	0.96	(0.68–1.34)
Q1-7. Explanation of treatment options and consultation	0.42	0.87	(0.63–1.21)
Q1-8. Satisfaction with treatment method	<0.001	1.97	(1.36–2.85)
Q1-9. Atmosphere conducive to asking questions	0.74	0.94	(0.63–1.40)
Q2. Talking with physician about symptoms and treatments related to urination at least once every 10 visits.	0.47	0.84	(0.52–1.35)
Age (≥70 years old)	0.86	1.03	(0.75–1.40)
Sex (female)	<0.001	1.89	(1.38–2.58)
Duration of medication exposure is ≥3 months.	0.06	1.38	(0.99–1.92)
Have not switched to another type of overactive bladder medication since starting to take it.	0.002	1.53	(1.16–2.01)

Chi-square probability: <0.001; Hosmer–Lemeshow *p*-value: 0.34; correct discrimination rate: 0.63. CI, confidence interval; OABSS, overactive bladder symptom score; OR, odds ratio.

**Table 4:** Stratified analysis in the discontinuation and continuation groups.

	Association with Medical Care Satisfaction (Endpoint 1)						Association with Effectiveness of OAB Medication * (Endpoint 2)					
	Discontinuation Group			Continuation Group			Discontinuation Group			Continuation Group		
Explanatory Variables	*p*-Value	Adjusted OR	95% CI	*p*-Value	Adjusted OR	95% CI	*p*-Value	Adjusted OR	95% CI	*p*-Value	Adjusted OR	95% CI
Q1-1. Medication’s effectiveness	<0.001	4.22	(2.00–8.92)	<0.001	8.43	(5.16–13.78)	-	-	-	-	-	-
Q1-2. Medication’s side effects	0.04	0.44	(0.20–0.97)	<0.001	0.34	(0.19–0.59)	-	-	-	-	-	-
Q1-3. Symptom enquiry	0.11	0.41	(0.14–1.21)	0.64	1.18	(0.59–2.36)	0.97	1.02	(0.42–2.43)	0.001	2.59	(1.46–4.61)
Q1-4. Explanation of treatment method	0.08	2.98	(0.88–10.07)	0.85	0.93	(0.47–1.85)	0.20	1.91	(0.71–5.15)	0.17	1.50	(0.84–2.67)
Q1-5. Explanation of effects	0.67	0.78	(0.25–2.43)	0.11	1.71	(0.88–3.32)	0.27	0.58	(0.22–1.53)	0.92	0.97	(0.55–1.72)
Q1-6. Explanation of side effects	0.006	3.47	(1.43–8.40)	0.08	1.65	(0.94–2.89)	0.78	1.12	(0.50–2.49)	0.86	1.04	(0.67–1.63)
Q1-7. Explanation of treatment options and consultation	0.09	2.12	(0.89–5.05)	0.38	1.27	(0.74–2.20)	0.32	1.47	(0.69–3.12)	0.21	0.75	(0.48–1.18)
Q1-8. Satisfaction with treatment method	0.86	1.08	(0.45–2.62)	<0.001	2.81	(1.55–5.09)	0.31	1.47	(0.70–3.09)	0.004	2.16	(1.28–3.64)
Q1-9. Atmosphere conducive to asking questions	0.08	2.57	(0.90–7.35)	0.01	2.32	(1.19–4.54)	0.77	1.14	(0.49–2.65)	0.14	0.65	(0.36–1.16)
Q2. Talking with physician about symptoms and treatments related to urination at least once every 10 visits	0.32	2.15	(0.48–9.65)	0.20	1.76	(0.74–4.18)	0.61	0.78	(0.30–2.03)	0.61	0.83	(0.41–1.69)
Age (≥70 years old)	0.26	0.61	(0.26–1.44)	0.31	1.33	(0.77–2.32)	0.76	1.12	(0.53–2.37)	0.68	1.09	(0.72–1.65)
Sex (female)	0.88	0.94	(0.40–2.22)	0.23	1.42	(0.81–2.50)	0.22	1.58	(0.76–3.27)	0.002	1.94	(1.27–2.95)
Duration of medication exposure is ≥3 months	0.11	2.07	(0.86–5.00)	0.07	0.53	(0.26–1.06)	0.32	1.45	(0.69–3.03)	0.002	2.30	(1.36–3.87)
Have not switched to another type of overactive bladder medication since starting to take it	0.48	1.31	(0.62–2.76)	0.04	1.65	(1.03–2.65)	0.63	0.86	(0.46–1.60)	0.006	1.65	(1.16–2.36)

* An improvement of ≥3 points in OABSS. CI, confidence interval; OAB, overactive bladder; OABSS, overactive bladder symptom score; OR, odds ratio.

## Data Availability

The datasets generated and/or analyzed during the current study are not publicly available due to the possibility of compromising research participant consent, but are available from the corresponding author on reasonable request.
